# Tandem Synthesis of Ultra-High Molecular Weight Drag Reducing Poly-α-Olefins for Low-Temperature Pipeline Transportation

**DOI:** 10.3390/polym13223930

**Published:** 2021-11-14

**Authors:** Ilya E. Nifant’ev, Alexander N. Tavtorkin, Alexey A. Vinogradov, Sofia A. Korchagina, Maria S. Chinova, Roman S. Borisov, Grigory A. Artem’ev, Pavel V. Ivchenko

**Affiliations:** 1 A.V. Topchiev Institute of Petrochemical Synthesis RAS, 29 Leninsky Pr., 119991 Moscow, Russia; tavtorkin@yandex.ru (A.N.T.); korchagina@ips.ac.ru (S.A.K.); chinova@yandex.ru (M.S.C.); borisov@ips.ac.ru (R.S.B.); phpasha1@yandex.ru (P.V.I.); 2 Chemistry Department, M.V. Lomonosov Moscow State University, 1–3 Leninskie Gory, 119991 Moscow, Russia; vinasora@gmail.com; 3 I.Ya. Postovsky Institute of Organic Synthesis, Ural Division of RAS, 22/20 Kovalevskoy Str., 620137 Yekaterinburg, Russia; griga1972@mail.ru

**Keywords:** chromium PNP catalysts, drag reducing, ethylene oligomerization, internal donor, linear α-olefins, branched α-olefins, polymerization, UHMW polyolefins, Ziegler–Natta catalysts

## Abstract

Ultra-high molecular weight poly-α-olefins are widely used as drag reducing agents (DRAs) for pipeline transportation of oil and refined petroleum products. The synthesis of polyolefin DRAs is based on low-temperature Ziegler–Natta (ZN) polymerization of higher α-olefins. 1-Hexene based DRAs, the most effective at room temperature, typically lose DR activity at low temperatures. The use of 1-hexene copolymers with C8–C12 linear α-olefins appears to offer a solution to the problem of low-temperature drag reducing. The present work aims to develop two-stage synthesis of polyolefin DRAs that is based on selective oligomerization of ethylene in the presence of efficient chromium/aminodiphosphine catalysts (Cr-PNP), followed by polymerization of the olefin mixtures, formed at oligomerization stage, using efficient titanium–magnesium ZN catalyst. We have shown that oligomerization of ethylene in α-olefin reaction media proceeds faster than in saturated hydrocarbons, providing the formation of 1-hexene, 1-octene, and branched C10 and C12 olefins; the composition and the ratio of the reaction products depended on the nature of PNP ligand. Oligomerizates were used in ZN polymerization ‘as is’, without additional treatment. Due to branched character of C10+ hydrocarbons, formed during oligomerization of ethylene, resulting polyolefins demonstrate higher low-temperature DR efficiency at low polymer concentrations (~1 ppm) in comparison with benchmark polymers prepared from the mixtures of linear α-olefins and from pure 1-hexene. We assume that faster solubility and more efficient solvation of the polyolefins, prepared using ‘tandem’ ethylene-based process, represent an advantage of these type polymers over conventional poly(1-hexene) and linear α-olefin-based polymers when used as ‘winter’ DRAs.

## 1. Introduction

The development of the energy-saving transportation and waste-free technologies are priority tasks for contemporary applied and fundamental science. To reduce pumping power and increase piping system capacity, modern pipeline transportation of crude oil and petroleum products use drag reducing agents (DRAs) comprised of ultra-high molecular weight (UHMW) polymers [1,2,3]. The drag reducing (DR) phenomena in dilute solutions of UHMW polymers was discovered by Toms [4] and subsequently studied by other scientists [5,6,7,8,9,10,11]. Hydrocarbon soluble UHMW poly-α-olefins are widely applied as DRAs [12,13,14]. Since the DR effect increases as the length of the macromolecule grows [14,15,16,17], the main task in the synthesis of poly-α-olefin DRAs is to achieve the highest possible degrees of polymerization (*DP*_n_).

Until quite recently, poly-α-olefin DRAs production used bulk polymerization of α-olefins, such as 1-hexene, 1-octene, 1-decene and 1-dodecene, in the presence of Ziegler–Natta catalysts [13]. Hydrocarbon solutions of UHMW polyolefins have an extremely high viscosity, the increase in viscosity during the reaction hampers α-olefin diffusion, hinders heat transfer and provokes a chain termination. As a result, in bulk polymerization the molecular weight of the polymer decreases at high monomer conversions [13,14,18,19,20], that is why the use of highly active and ultra-dispersed ZN catalysts and subzero reaction temperatures are required.

At room temperatures, UHMW poly(1-hexene) represent a best choice among other poly-α-olefin DRAs due to the highest mass efficiency (the longest polymer chain for equal MW) and satisfactory solubility in hydrocarbons; homopolymers of higher α-olefins (1-octene, 1-decene, etc.) have substantially lower DR efficiency in comparison with poly(1-hexene) [21,22]. However, the DR efficiency of poly(1-hexene)s is falling rapidly with the decreasing temperature of the hydrocarbon fluid [23]. As mentioned in the patent literature [24,25,26], the formulations of “winter” DRAs are based on copolymers of 1-hexene with higher α-olefins. 

**Scheme 1:**
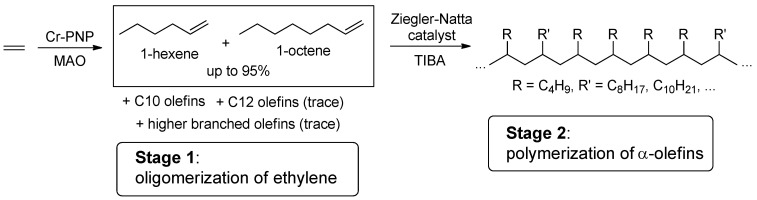
Tandem ethylene-based approach to UHMW poly-α-olefins.

To a first approximation, the products of selective trimerization of ethylene, catalyzed by aminodiphosphine chromium complexes (Cr-PNP), represent comonomer mixtures with desirable 1-hexene/α-olefin ratios [27,28] (Scheme 1). Oligomerization of ethylene, catalyzed by Cr-PNP, was previously conducted in saturated hydrocarbons or in aromatic solvents [29,30,31,32,33,34]. We proposed that the use of the α-olefins instead of conventional hydrocarbon solvents at the stage of ethylene oligomerization will provide 1-hexene enriched mixtures of terminal olefins that can be used in the synthesis of UHMW polymers ‘as is’, without any pre-treatment.

In the present work, we found that the activity of Cr-PNP catalysts in α-olefins is substantially higher than in saturated hydrocarbons. By the use of two Cr-PNP catalysts with different C6/C8 selectivity, we prepared the mixtures of the oligomers, analyzed their composition, and synthesized UHMW polyolefins. Copolymers were also prepared from the mixtures of linear α-olefins with the same comonomer ratios. The results of the comparative studies of the DR efficiency of copolymers and benchmark poly(1-hexene) having the same MW are also presented and discussed.

## 2. Materials and Methods

### 2.1. Solvents and Reagents

Most of the solvents and chemicals were supplied by Merck (Darmstadt, Germany). 1-Hexene (97%), 1-octene (98%), 1-decene (94%), 1-dodecene (95%), *n*-pentane (95%) and *n*-heptane (95%) were dried over sodium and distilled before use under argon atmosphere. Toluene (99.5%), diethyl ether (Et_2_O, ≥99%) and tetrahydrofuran (THF, ≥99%) were refluxed over sodium/benzophenone ketyl and distilled. TiCl_4_ (99.5%) was distilled before use and stored in polytetrafluoroethylene (PTFE) capped vessels. Ethanol (EtOH, ≥99%) was refluxed over Mg turnings (≥99.8%) and distilled. Dimethyl sulfate (≥99%), methyl iodide (99%), formaldehyde (37% aq. solution), 2-ethylhexanal (96%), NH_4_Cl (≥99.5%), dimethyl sulfoxide (DMSO, ≥99.7%), hexadecyltrimethylammonium chloride (50% solution in 2-propanol/water 3:2), triisobutylaluminium (TIBA, 1M solution in hexane), trimethylaluminium (TMA, 2M solution in toluene), modified methylalumoxane (MMAO-12, 1.2M solution in toluene), chromium (III) acetylacetonate (Cr(acac)_3_, 99.99% trace metal basis), and methanol (99.8%) were used as purchased. Ethylene (99.999%, Linde Gas, Russian Federation) was used without purification.

Diphosphine ligands N-(bis(2-methoxyphenyl)phosphino)-1,1-bis(2-methoxy-phenyl)-N-methylphosphinamine (**L1**) [30] and 5-(bis(2-methoxyphenyl)-phosphino)-6-(2-methoxyphenyl)-5,6-dihydrodibenzo[*c*,*e*][1,2]azaphosphinine (**L2**) [35] were synthesized according to the published procedures. 

### 2.2. Analysis

The ^1^H and ^13^C {^1^H} NMR spectra were recorded on a Bruker AVANCE 400 spectrometer (400 MHz, Bruker Corporation, Billerica, MS, USA) at 20 °C (organic compounds) and at 50 °C (polymer samples). CDCl_3_ (D 99.8 %, Cambridge Isotope Laboratories, Inc., Tewksbury, MS, USA) was used as purchased. Polymer solutions for spectral study were prepared by 6–12 h dissolution of polymer samples (3–5 mg) in CDCl_3_ (0.7 mL) at 50 °C with stirring. The chemical shifts are reported relative to the solvent residual peaks (δ = 7.26 ppm for ^1^H and 77.16 for ^13^C {^1^H} NMR spectra). 

Elemental analysis (C, H, N, O) was performed using a Perkin Elmer Series II CHNS/O Analyzer 2400 (Perkin Elmer, Waltham, MS, USA).

GC analysis was performed on a KRISTALL-2000M gas chromatograph (Chromatec, Yoshkar-Ola, Russian Federation) equipped with a SolGel-1ms (60 m × 0.25 mm × 0.25 μm) column and a flame ionization detector. Helium was used as the carrier gas at the rate of 1.364 cm^3^/min and with the split ratio of 73.3: 1. The injection temperature was 250 °C, and the column temperature was 100 °C within 5 min before increasing from 100 °C to 300 °C at a rate of 10 °C/min.

GC×GC/MS-FID analysis was performed using Leco Pegasus^®^ BT 4D instrument (LECO Corp., St. Joseph, MI, USA) equipped with Agilent 7890A gas chromatograph (Agilent Technologies, Santa Clara, CA, USA) with an embedded second oven, a two-stage cryomodulator, a flow splitter, a time-of-flight mass analyzer and a flame ionization detector. The gas chromatographic system included two columns (the first was Restek Rxi-5Sil 30 m × 0.25 mm × 0.25 μm, the second Restek Rxi-17Sil 1.7 m × 0.10 mm × 0.10 μm, Restek, Centre County, PA, USA) and was operated in temperature–programmed mode (initial temperature 40 °C for two minutes, heating to 320 °C at a rate of 10 °C/min, holding for 5 min, the temperature of the second oven and the modulator was maintained at a level of 10 and 20 °C higher than the temperature of the first oven, respectively) using helium as a carrier gas. The MS instrument was operated in 70 eV electron ionization mode using 100 Hz recording mode. The flame ionization detector temperature was 340 °C, hydrogen and air flow rates were 40 cm^3^/min and 450 cm^3^/min, respectively. Analysis results were processed using the CromaTOF software (LECO Corp., St. Joseph, MI, USA), the analytes were identified using NIST 20 database (National Institute of Standards and Technology, Gaithersburg, MD, USA), the relative quantities were determined on the basis of FID data presuming equal response factors for all olefins.

Size exclusion chromatography (SEC) measurements were performed in THF (40 °C, flow rate 1 mL/min) using an 1260 Infinity II (Agilent Technologies, Santa Clara, CA, USA) integrated instrument equipped with a PLgel MIXED-C column (2 × 10^2^–2 × 10^6^ Da), an autosampler, and an refractive index detector. The measurements were recorded with universal calibration according to a polystyrene standard.

### 2.3. Oligomerization Experiments

Oligomerization experiments were conducted according to the method reported previously [35] in a Polyclave (Büchi AG, Uster, Switzerland) autoclave equipped with 1 L stainless steel reactor and fluidized jacket, connected to a thermostatic bath. Ethylene was added to the reaction through a mass flow controller. Solvents and reagents were introduced into the reactor using burettes, and a screw-type syringe was used for the injection of the catalyst solutions. The ethylene uptake data were recorded, along with the reactor pressure, and the reaction mixture temperature.

In a typical experiment, the rigorously cleaned autoclave was heated to 120 °C under vacuum for 60 min, and then cooled to reaction temperature and back-filled with ethylene (10 bar), which was then vented to 1 bar by septa to purge the inlet valve. Solvent (*n*-heptane or α-olefin, 120 mL) and TMA (1.5 mL of 2M solution in toluene) were then added using an injection system. The autoclave was pressurized with ethylene to 10 bar, vented, pressurized to 30 bar, and heated to 50 °C. On a Schlenk line, a catalyst solution was prepared by the reaction of Cr(acac)_3_ (30 μmol) with a PNP ligand (40 μmol) in toluene (4 mL), followed by the addition of MMAO-12 (5 mL, 1.2 M solution in toluene, Al/Cr = 200), and then stirred for 15 min. The catalyst was transferred to the autoclave by syringe. The pressure was maintained at a constant value throughout the reaction by the continuous addition of ethylene, which was monitored using flowmeter. After a defined period of time, the gas supply was closed and the reactor cooled to 5 °C and carefully vented. The data on oligomerizate composition are presented and discussed in Section 3.1. The products of oligomerization with different hexene-1/octene-1 ratios **M1** and **M2** were selected for polymerization experiments. These samples were obtained using Cr-PNP catalysts, based on PNP ligands **L1** and **L2**, respectively, in 1-hexene reaction media.

### 2.4. Synthesis of New Internal Donor, 3,3-Bis(methoxymethyl)heptane (BMMH)

#### 2.4.1. 2-Butyl-2-ethylpropane-1,3-diol

2-Ethylhexanal (120 g, 0.920 mol) and formaldehyde (155 g of 37% aq. solution, 2.350 mol) were placed into 500 mL flask. The solution of NaOH (92.7 g, 1.110 mol) in H_2_O (100 mL) was added dropwise with variable rates (0.100 mol/h during the first two hours, 0.150 mol/h during 3rd and 4th hours, and 0.220–0.250 mol/h in the time remaining). The mixture was neutralized to neutral pH by the addition of conc. H_2_SO_4_ with stirring and intensive cooling. The organic layer was separated and distilled under reduced pressure (B. p. 130–135 °C/8 mbar). The yield 133.0 g (90%). 

#### 2.4.2. 2-Ethyl-2-(methoxymethyl)hexan-1-ol

2-Butyl-2-ethylpropane-1,3-diol (110 g, 0.690 mol) was added dropwise at 60 °C to the solution of NaOH (70 g, 1.750 mol) in H_2_O (70 mL). The mixture was cooled to 40–45 °C, and dimethyl sulfate (95 mL, 1 mol) was added within 2 h, maintaining a temperature of 45–50 °C. After 1 h of stirring, H_2_O (200 mL) and hexane (100 mL) were added, organic layer was separated, aqueous layer was washed by hexane (20 mL). Combined organic phase was washed by water (2 × 20 mL) and evaporated under reduced pressure. The residue was distilled collecting the fraction 90–110 °C/16 mbar. The yield 84.0 g (69.9%), colorless liquid containing ~10% of BMMH (for ^1^H NMR spectrum, see Figure S1 in the Supplementary Materials). The synthesis was repeated twice, the yields were 83.5 and 83.8 g (69.4 and 69.7%, respectively). Distillation residues were combined and dissolved in benzene (100 mL). Solution of NaOH (40 g) in H_2_O (40 mL), and hexadecyltrimethylammonium chloride solution (0.5 mL) were added. Dimethyl sulfate (53 mL, 0.550 mol) was added dropwise, maintaining a temperature of 45–50 °C. After 1 h of stirring, the mixture was treated as described above, and additional amount of 2-ethyl-2-(methoxymethyl)hexan-1-ol (37.4 g) was obtained by distillation. The total yield of the product was 80%.

#### 2.4.3. 3,3-Bis(methoxymethyl)heptane (BMMH)

NaH (60% suspension in mineral oil, 25.0 g, 0.625 mol) was placed in 500 mL flack, washed by hexane (3 × 50 mL), and THF (200 mL) was added. The mixture of 2-butyl-2-ethylpropane-1,3-diol (104 g, 0.600 mol) and methyl iodide (38.0 mL, 0.61 mol) was added dropwise (the reaction begins after the addition of ~5 mL of the mixture). After a slowdown of the release of hydrogen, the addition was continued with external cooling of the mixture (~4 h). After conditioning during 12 h, H_2_O (10 mL) was added with caution. Organic phase was separated, the precipitate was dissolved in H_2_O (25 mL), the solution was washed by hexane (20 mL). Combined organic phase was washed by water (2 × 20 mL) and evaporated under reduced pressure. The residue was distilled collecting the fraction 95–100 °C/13 mbar. The yield 107 g (95%), colorless liquid. Elemental Analysis: for C_11_H_24_O_2_ calculated (%): C, 70.16; H, 12.85; O, 16.99; found (%): C, 70.09; H, 12.89; O, 17.02. ^1^H NMR (400 MHz, CDCl_3_, 20 °C) δ: 3.29 (s, 6H); 3.14 (s, 4H); 1.26 (q, ^3^*J* = 7.5 Hz, 4H); 1.18 (m, 4H); 0.88 (t, ^3^*J* = 7.2 Hz, 3H); 0.78 (t, ^3^*J* = 7.5 Hz, 3H). ^13^C {1H} NMR (101 MHz, CDCl_3_, 20 °C) δ: 75.54; 59.34; 40.94; 31.03; 25.13; 24.05; 23.74; 14.29; 7.46. NMR spectra of BMMH are presented in Figures S2 and S3 in the Supplementary Materials.

### 2.5. Synthesis of Titanium–Magnesium Catalyst

Titanium–magnesium catalyst (TMC) was obtained using the method reported previously [36] (type C2 catalyst in this article) with the replacement of the BMMP donor by BMMH donor and with substantial modifications of the experimental protocol. For details of the synthesis and analysis of TMC, see Section S1 in the Supplementary Materials.

### 2.6. Polymerization Experiments

#### 2.6.1. Preparation of Model Mixtures of Linear α-Olefins

Model mixtures **M1c** and **M2c** were prepared in a glove box by weighting of the calculated amounts of 1-hexene, 1-octene, 1-decene, 1-dodecene and toluene that correspond to the ratios of 1-hexene, 1-octene, C10, C12 olefins, and toluene in oligomerizates **M1** and **M2**, respectively.

#### 2.6.2. Low-Temperature Polymerization

1-Hexene, oligomerization product, or model mixture of linear α-olefins (20 mL) was placed into 50 mL vial, TIBA (0.4 mL of 1M solution in heptane) was added. The vial was cooled to –40 °C, TMC suspension (8 μL, [Ti] = 0.16 M) was added with stirring, and the mixture was stored at –12 °C within 10 days. The yields and characteristics of polymers **PH**, **P1**, **P1c**, **P2**, and **P2c** are presented and discussed in Section 3.3 and in Section S2 in the Supplementary Materials.

### 2.7. Drag Reducing Experiments

#### 2.7.1. Sample Preparation

The sample of polymer (0.250 g) was dissolved with slow stirring (magnetic stirrer, 60 rpm) in *n*-heptane (200 g) within 24 h. The resulting viscous solutions having 1250 ppm concentration (1 g) was taken and diluted by *n*-heptane (49 g) yielding 25 ppm solution that was used for drag reducing tests after appropriate dilution.

#### 2.7.2. Capillary Turbulent Rheometry

Drag reducing tests were performed using turbulent capillary rheometer (Reynolds number 1 × 10^4^ for *n*-heptane) by flowing the samples of the polymer solution and pure solvent at given temperature and fixing expiration times for the solvent and polymer solutions. Drag reducing (DR) was calculated by the Equation (1).

(1) $$DR=\frac{\lambda_{0}-\lambda_{p}}{\lambda_{0}}\times 100\%=\frac{t_{0}^{2}-t_{p}^{2}}{t_{0}^{2}}\times 100\%$$

where *λ*_0_—the hydrodynamic drag coefficient of the solvent; *λ_p_*—the hydrodynamic drag coefficient of the polymer solution; *t*_0_—expiration time for the solvent; *t_p_*—expiration time for polymer solution. The test was carried out three times for each sample of the polymer solution.

## 3. Results and Discussion

### 3.1. The Main Idea of the Two-Stage Synthesis of Polyolefin DRAs

**Scheme 2:**
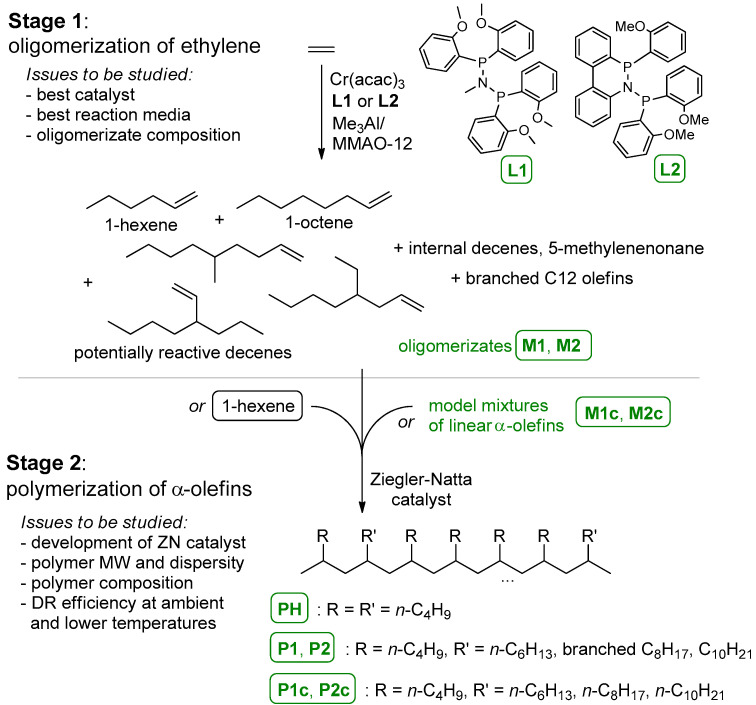
Ethylene-based synthesis of polyolefin DRAs, studied in this work: scope and content.

Selective oligomerization of ethylene using chromium-diphosphine catalysts seems to be the most efficient synthetic approach to 1-hexene, 1-octene or 1-hexene/1-octene mixtures [30,37,38,39,40,41]. Logically, this reaction was used in tandem production of LLDPE, copolymers of ethylene and 1-hexene (or 1-octene) [42,43]. However, metallacyclic mechanism of ethylene oligomerization when using Cr-PNP catalysts [30,32,44,45,46,47,48] allows for the formation of significant amounts of C10 and C12 hydrocarbons via coordination/insertion of 1-hexene or 1-octene molecules instead of ethylene molecule in the course of the reaction. As a result, C10 (Scheme 2) and C12 olefins of *branched* molecular structure are formed [27,28]. The presence of higher branched α-olefins allows us to consider such oligomerizates as prospective comonomer feedstocks for UHMW polyolefins. Therefore, two-stage ethylene-based synthesis of polyolefin DRAs (Scheme 1 and Scheme 2) seemed feasible.

### 3.2. Oligomerization of Ethylene

#### 3.2.1. The Use of α-Olefins as a Reaction Media 

To assess the feasibility of this two-stage approach to polyolefin DRAs, we selected two diphosphine ligands, **L1** [30] and **L2 [35]** (Scheme 2) for the preparation of Cr-PNP catalysts of the oligomerization stage, **Cr-L1** and **Cr-L2**, respectively. Hundreds of chromium diphosphine complexes have been explored to date [32,34,37,38,39,40,41], but high (TOF up to 10^6^ h^−1^) and steady catalytic activity was demonstrated by one of the first Cr-PNP catalysts of selective trimerization of ethylene, **Cr-L1** (Scheme 2) [30]. Recently we proposed novel type of ‘constrained’ diphosphine ligands, containing 5,6-dihydrodibenzo[*c*,*e*][1,2]azaphosphini-ne fragment, and demonstrated high activity of Cr-PNP catalyst **Cr-L2** (Scheme 2) in oligomerization of ethylene [35]. 

In our previous comparative studies [35], we prepared **Cr-L1** and **Cr-L2** by the reaction of Cr(acac)_3_ with diphosphine ligands, followed by activation using MMAO-12 and TMA. Both catalysts demonstrated close activities; however, **Cr-L2** catalyzed tri/tetramerization with a formation of 1-hexene and 1-octene in ~2:1 ratio by weight. In addition, both oligomerizates contained C10 and C12 olefins in visible amounts. Therefore, it seemed appropriate to use the products of ethylene oligomerization as a monomer mixture for the further polymerization in the presence of titanium–magnesium ZN catalyst.

However, there are two factors that complicate the immediate use of such oligomerizates in ZN polymerization. First, organoaluminum activators MMAO-12 and TMA are not consistent with ZN catalysts; the latter are activated preferably with TIBA. Therefore, minimal amounts of MMAO-12 and TMA should be used at the first stage. Second, even with the high activity of **Cr-L1** and **Cr-L2**, the resulting reaction mixtures contain moderate (10–15%) amounts of the inert solvents [35], which is completely unnecessary at the further polymerization stage due to polymer swelling. 

**Figure 1:**
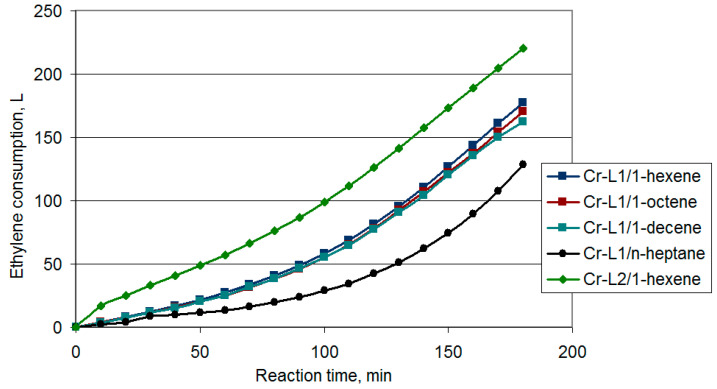
Ethylene consumption curves for ethylene oligomerization using Cr-PNP catalysts (Cr(acac)_3_ (30 μmol)/ PNP ligand (40 μmol)) activated by MMAO-12 at [Al]/[Cr] ratio of 200, 120 mL of the solvent, 30 bar, 50 °C).

In all experiments on Cr-PNP catalyzed oligomerization of ethylene described in the literature, the choice of the reaction media was limited by saturated hydrocarbons and aromatic solvents [38,41,49,50,51]. Given that α-olefins are *formed* during ethylene oligomerization, we proposed that α-olefins can be *initially* used as a reaction media, and made comparative experiments on ethylene oligomerization, catalyzed by **Cr-L1**, in *n*-heptane, 1-hexene, 1-octene, and 1-decene. In addition, we studied an opportunity to reduce the content of MMAO-12 and TMA (that may hamper further polymerization) and found appropriate [Al]/[Cr] ratio of 200 for oligomerization stage. The results of our experiments under optimized conditions are presented visually in Figure 1.

As can be seen in Figure 1, **Cr-L1** catalyzed oligomerization of ethylene in α-olefin media proceeds considerably faster in comparison with oligomerization in *n*-heptane, the latter had been characterized by longer induction period. The rate of the reaction was essentially independent of the type of α-olefin used (although the activity slightly decreased when moving from 1-hexene to 1-decene). Since 1-hexene is the most affordable and accessible higher α-olefin, the experiment on **Cr-L2** catalyzed oligomerization of ethylene was carried out in 1-hexene media, and **Cr-L2** catalyst demonstrated much more higher activity in comparison with **Cr-L1**. The reaction products, obtained during ethylene oligomerization in 1-hexene, catalyzed by **Cr-L1** and **Cr-L2**, have been chosen for the further studies of copolymerization as comonomer mixtures **M1** and **M2**, respectively. 

#### 3.2.2. Composition and Molecular Structure of the Oligomerization Products

The mechanism of ethylene oligomerization when using Cr-PNP catalysts [30,32,44,45,46,47,48] allows for the formation of C10 and C12 hydrocarbons via coordination/insertion of 1-hexene or 1-octene molecules instead of ethylene molecule in metallacyalic reaction intermediates. For C10 oligomerization products branched molecular structure was proposed and proved (Scheme 2, Table 1) [27,28]. 

We made GC×GC/MS-FID analysis of the oligomerizates **M1** and **M2**, selected for the further polymerization experiments (Table 1). The results of this analysis were in line with the data reported previously [27]. In particular, the main C10 component in oligomerizates **M1** and **M2** was 5-methyl-1-nonene (4.5 and 9.6 wt.%, respectively). The combined share of two other marked components, 4-vinyloctane and 4-ethyl-1-octene, was found to be roughly the same. These products represent α-olefins that are potentially reactive in ZN polymerization. The relative amounts of 1-decene in oligomerizates **M1** and **M2** were negligible, and the total relative content of the three remaining C10 reaction products, namely, 5-methylenenonane, 4-decene, and 5-decene, was found to be ~0.5 wt.%. Therefore, both oligomerizates **M1** and **M2** in their composition mainly contained α-olefins potentially reactive in ZN polymerization.

### 3.3. Optimization and Preparation of Titanium–Magnesium ZN Catalyst (TMC)

#### 3.3.1. Synthesis of Internal Donor BMMH 

In our previous study, we showed that titanium–magnesium ZN catalyst (TMC), prepared by the reaction of Mg(OEt)_2_ with TiCl_4_ in toluene [52] in the presence of the latest generation of diether donor, 3,3-bis(methoxymethyl)pentane (BMMP) [36,53], demonstrated the best characteristics in α-olefin polymerization [36]. However, the synthetic availability and cost of BMMP, synthesized from 2,2-diethylpropane-1,3-diol, focused us on the search of the more synthetically accessible donors. As a result of our studies, an alternative donor with the same characteristics, namely, 3,3-bis(methoxymethyl)heptane (BMMH), had been chosen. This donor was previously used in propylene polymerization [54,55] but still had not been appropriately characterized as new organic compound, as it is in the present paper (see Section 2.4.2). 

**Scheme 3:**
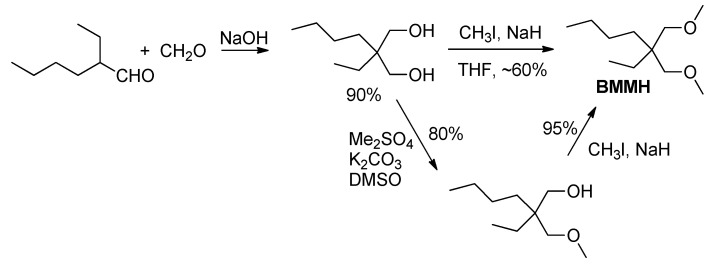
Synthesis of internal donor 3,3-bis(methoxymethyl)heptane (BMMH).

The synthesis of BMMH is based on the reaction of 2-ethylhexanal with formaldehyde (aldol and Cannizzaro reactions) followed by alkylation in a similar way to the method proposed by Agnes and Borsotti [56,57]. The yield of the intermediate diol was almost quantitative; however, double alkylation failed (~60% yield), and the total yield of 76% was obtained when two-stage alkylation was used (Scheme 3).

#### 3.3.2. Synthesis of TMC 

In our further development of TMC, targeted on the synthesis of α-olefin copolymers, we explored the potential of the method reported in [36]. This method was based on the addition of TiCl_4_ and then BMMP to suspension of Mg(OEt)_2_. To obtain the catalyst with highest productivity, we studied the sequence of the reagent mixing, and found that the most active TMC can be obtained by the addition of the Mg(OEt)_2_ suspension to excess of TiCl_4_, followed by the addition of BMMH. For details of the experiments on optimization of the synthesis of TMC and comparative catalytic experiments, see Section S1 in the Supplementary Materials. 

### 3.4. Polymerization and Polymer Analysis

#### Polymerization Experiments

To prepare benchmark UHMW poly(1-hexene), we carried out bulk polymerization of 1-hexene, containing calculated amount of toluene, at –12 °C. After 10 days, 97% conversion was detected with the obtaining of homopolymer **PH**. This method was also used in the synthesis of copolymers **P1**–**P2c**, and due to high activity of TMC (see Section S1.3 in the Supplementary Materials), high polymer yields were achieved after 10 days (Table 2).

Benchmark poly(1-hexene) (**PH**) and copolymers (**P1**, **P1c**, **P2**, **P2c**) had *M*_p_ in the range 4.2–5.5 × 10^6^ Da. The values of *M*_n_ (3.2 × 10^6^ Da for **P1** and ~2 × 10^6^ Da for other copolymers) along with narrow MWD (*Ð*_M_ = 1.6–2.9) allowed for the consideration of all copolymers obtained as UHMW polyolefins suitable for DR studies.

Lower yields of **P1** and **P2** in comparison with copolymers of linear α-olefins **P1c** and **P2c** can be attributed to the presence of inactive olefins (5-methylenenonane, internal decenes) and low-active α-olefins (4-vinyloctane, 4-ethyl-1-octene) in starting oligomerizates **M1** and **M2**. The presence of these olefins was detected by ^1^H NMR spectroscopy of the final reaction mixtures (see Figures S5 and S7 in the Supplementary Materials).

**Figure 2:**
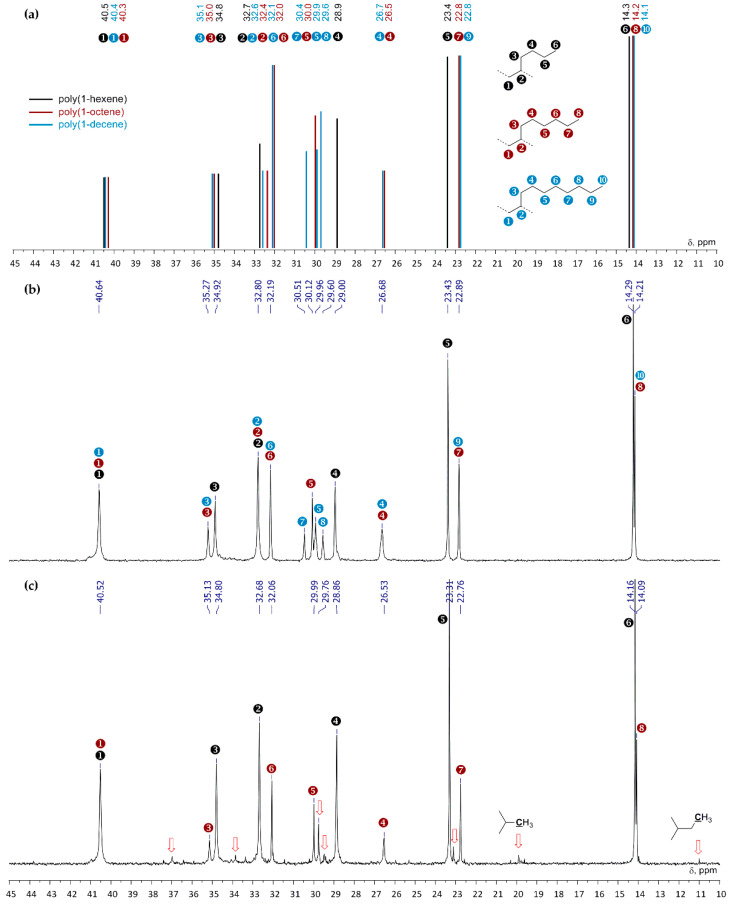
(**a**) Schematic reference ^13^C {^1^H} NMR spectra of isotactic poly(1-hexene), poly(1-octene), and poly(1-decene) (^13^C {^1^H} NMR spectra of these homopolymers are presented in Figure S4 in the Supplementary Materials); ^13^C {^1^H} NMR spectra (CDCl_3_, 50 °C, 101 MHz) of the copolymers **P2c** (**b**) and **P2** (**c**). New signals in spectrum of **P2** are pointed by red arrows.

^13^C {^1^H} NMR spectra of **P1** and **P2** have confirmed insertion of branched decenes. Comparison of the spectrum of **P2c** (Figure 2b, with identification of all signals using schematic reference spectra of isotactic poly(1-hexene), poly(1-octene) and poly(1-decene), presented in Figure 2a) with the spectrum of **P2** (Figure 2c) demonstrates the absence of the signals of poly(1-decene) in the spectrum of **P2**. However, the spectrum of **P2** contained marked characteristic signals of >CHCH_3_ groups (δ = 20 ppm) and subtle signal of >CHCH_2_CH_3_ groups (δ = 11 ppm) related to the products of the insertion of 5-methyl-1-nonene and 4-ethyl-1-octene, respectively. The signal of >CHCH_3_ groups (δ = 20 ppm) can also be seen in ^13^C {1H} NMR spectrum of **P1** (see Figure S9 in the Supplementary Materials). ^13^C {^1^H} NMR spectra of **P1** and **P2** (see Section S2 in the Supplementary Materials) revealed a number of low-intensity signals, related to the products of the insertion of branched C10 α-olefins. 

However, the relative intensities of these new signals were substantially lower than the signals of the fragments, related to 1-decene insertion, in the spectra of copolymers **P1c** (Figure 2b) and **P2c**. In this way, the insertion of branched C10 α-olefins seemed incomplete. This latter conclusion was of particular interest focusing attention on the impact of the *lower* amounts of *branched* higher α-olefins on DR efficiency of the copolymers.

### 3.5. Drag Reducing Efficiency

**Figure 3:**
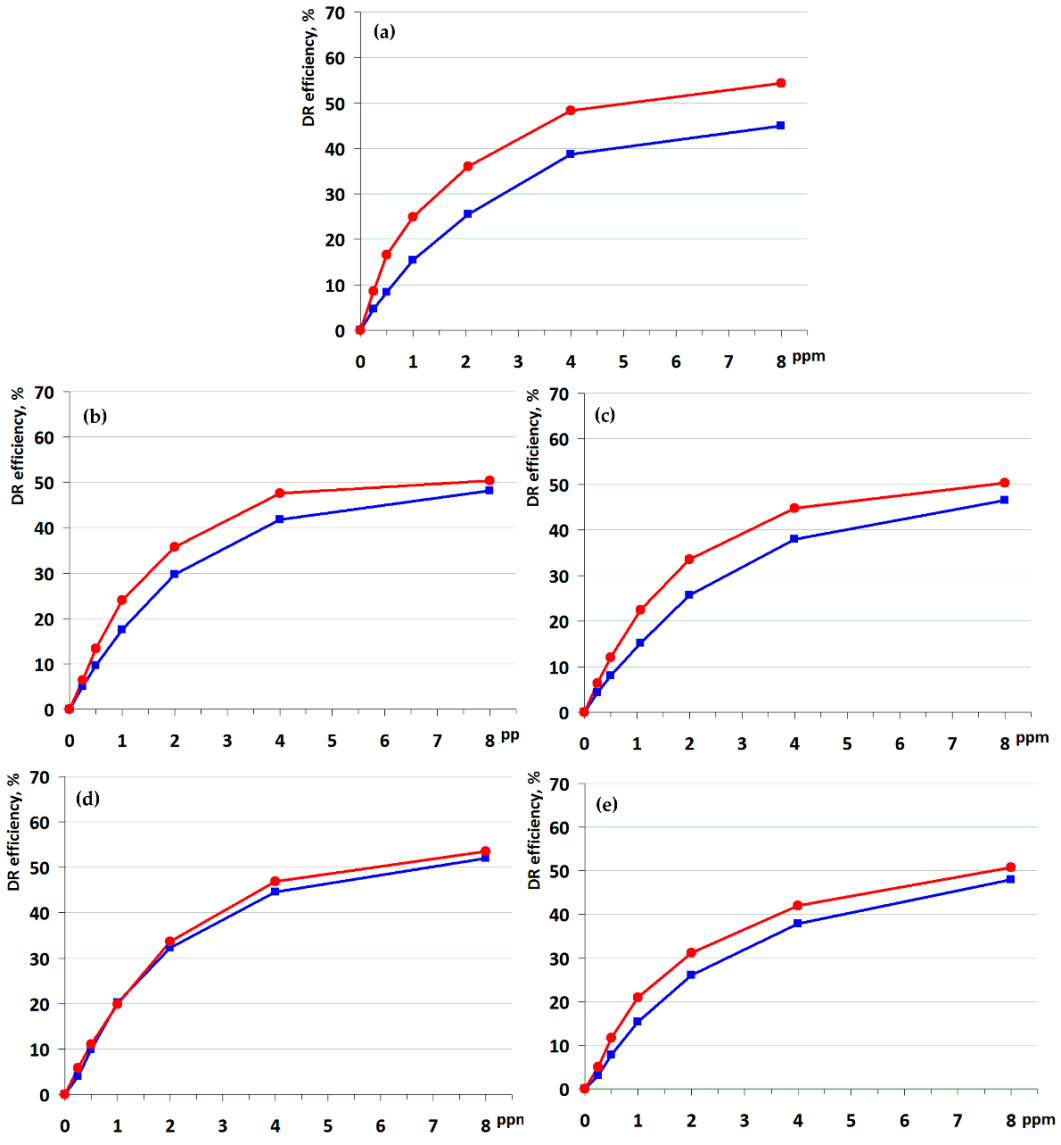
Dependence of the DR effect on the concentration for solutions of polymers **PH** (**a**), **P1** (**b**), **P1c** (**c**), **P2** (**d**), and **P2c** (**e**), in *n*-heptane at 7 °C (blue lines) and at 22 °C (red lines). Capillary turbulent flow rheometry data, the average of the three measurements for each point.

Polymer samples, obtained by bulk polymerization, dissolve slowly in aliphatic hydrocarbons. Since intensive mixing can lead to the destruction of macromolecules and a decrease in the drag reducing effect, polymer solutions in *n*-heptane were made by dissolving polymer samples with slow stirring for 1 day. Various experimental methods can be used to investigate the drag reducing effect, including rotational rheometry at high Reynolds numbers [1,9,58,59,60] and turbulent flow rheometry [14,61]. In the present work, comparative studies of the DR efficiency of polymers **PH**–**P2c** were carried out at 7 and 22 °C using turbulent capillary rheometry [14] of heavily diluted polymer solutions (see Section 2.6). The chosen test temperatures correspond to average temperatures of oil and petroleum products during pipeline transportation in the winter and summer seasons, respectively [23]. The results of the measurements as dependences of DR on the polymer concentration for the second passage of the DRA solution through the rheometer tube are presented in Figure 3.

As can be seen in Figure 3, poly(1-hexene) **PH**, as well as copolymers **P1** and **P1c**, demonstrated lower DR effect at lower temperature. For copolymers **P2** and **P2c**, DR curves for different temperatures have been found to be closely aligned. DR efficiencies of all pre-prepared polymer solutions were close for 4–8 ppm concentrations. Perhaps the most significant observation was the highest DR efficiency of the copolymer **P2** at 7 °C in 1 ppm concentration (20% vs. ~15% for all other polymers). The value of DR of 20% is characteristic for real pipeline transportation of light petroleum products using DRA dispersions; however, poly(1-hexene) DRAs fail to reach even 10% efficiency at 5–7 °C [23]. 

We think that the difference in low temperature DR efficiency of **P2** and other polymers can be attributed to more efficient solvation of **P2**. Based on acoustic measurements of the solvation numbers and of DR efficiency, Brostow et al. have shown that the solvation numbers go symbiotically with that efficiency [7]. In other words, the solvated polymer domains in good solvents are larger and, therefore, more effective as DRAs. The effect of the temperature on DR efficiency of polyolefin DRAs can be explained by this factor: more regular poly(1-hexene) **PH** as well as 1-hexene enriched copolymers **P1** and **P1c** are poorly solvated at lower temperatures, while copolymer **P2**, which has a less ordered microstructure due to the presence of the higher content of branched alkyl groups, retains its capacity for solvation.

The effectiveness of DRAs in real-world operation is determined by the following factors: the value of DR efficiency, the rate of solubility to achieve efficient concentration of DRA, and the rate of polymer degradation. Increasing the length of the polymer chain results in an increase inboth DR effect [14,15,16,17,62] and polymer degradation rate [63,64,65], and decreases the rate of solubility [14]. As was shown by Moussa and Tiu [66], most of the degradation takes place at the region of the entrance of DRAs because of the high extensional straining of polymer molecules; therefore, slow solubility and limited solvation increase the probability of the polymer chain scission.

The results of our comparative study of the DR efficiency of poly(1-hexene) **PH**, 1-hexene enriched copolymers **P1** and **P1c**, and copolymers, containing substantial amounts of 1-octene and decenes **P2** and **P2c**, allow us to explain the loss of the DR efficiency of poly(1-hexene) DRAs at low temperature by lower dissolution/solvation rates along with relatively high degradation rates—with the result that efficient concentration of UHMW additive is simply not reached. In this way, copolymers **P1** and **P2** that represent the products of tandem, ethylene-based process, seem more prospective as low-temperature polyolefin DRAs.

## 4. Conclusions

In the present work, we have demonstrated the principle possibility of the ‘tandem’ synthesis of UHMV polyolefin DRAs by ethylene oligomerization, catalyzed by Cr-PNP complexes, followed by low-temperature polymerization of the oligomerizate, catalyzed by efficient titanium–magnesium ZN catalyst. For the oligomerization stage, we have shown the preference of the use of α-olefins as a reaction media. In particular, catalyst **Cr-L1** demonstrated higher activities in α-olefins compared with *n*-heptane. In addition, the oligomerizates contained minimal amounts of inert solvents, thus providing their use as a comonomer feed for subsequent polymerization without any additional treatment.

Two oligomerizates **M1** and **M2** were synthesized in 1-hexene using highly selective trimerization catalyst **Cr-L1** and tri/tetramerization catalyst **Cr-L2**. GC analysis of the oligomerizates showed the presence of branched C10 α-olefins. UHMW polyolefins were obtained from oligomerizates **M1** and **M2**, model mixtures of linear α-olefins **M1c** and **M2c**, and 1-hexene. During the study of DR efficiency of copolymers we found that copolymer **P2**, obtained by tandem process using **Cr-L2** catalyst at oligomerization stage, had the highest value of low-temperature DR efficiency at low concentrations (~1 ppm). We propose that this was due to irregular microstructure of **P2**, the presence of C6 and branched C8 side chains, resulting in an increase in dissolution rate and solvation number. We propose that low efficiency of poly(1-hexene) DRAs at low temperatures can be attributed to inaccessibility of minimum essential polymer concentrations due to low solubility/solvation rates and relatively high degradation rates. 

Therefore, the product of the tandem process represent the best choice among UHMW hydrocarbon-soluble polymers for low-temperature transportation of light petroleum products. Conditions are thus created for the further research and development of ethylene-based technology of the production of ‘winter’ poly-α-olefin DRAs.

## Figures and Tables

**Table 1:** Compositions of the oligomerization products, model linear α-olefin mixtures, and 1-hexene benchmark feedstock.

Number of C Atoms	Structure	Name	wt.% of the Components				
			H	M1	M1c	M2	M2c
6	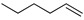	1-hexene	97.9	74.4	74.4	47.2	47.2
7	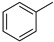	toluene	2.1	2.1	2.1	2.1	2.1
8	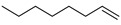	1-octene	–	10.4	10.4	25.1	25.1
10	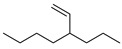	4-vinyloctane	–	2.9	–	5.1	–
10	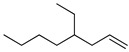	4-ethyl-1-octene	–	2.1	–	3.9	–
10	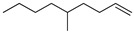	5-methyl-1-nonene	–	4.5	–	9.6	–
10	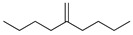	5-methylenenonane	–	1.6	–	1.9	–
10	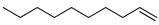	1-decene	–	0.2	11.6	0.2	21.0
10	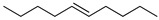	5-decene	–	0.1	–	0.1	–
10	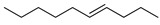	4-decene	–	0.2	–	0.2	–
12	–	C12 alkenes ^1^	–	1.5	1.5 ^2^	4.6	4.6 ^2^

^1^ A complex mixture of the isomers. ^2^ 1-Dodecene.

**Table 2:** The results of polymerization experiments.

Polymer	Yield, %	*M*_p_, ×10^6^ Da ^1^	*M*_n_, ×10^6^ Da ^1^	*Ð*_M_^1^	Comonomer Molar Ratio ^2^		
					C6	C8	C10
**PH**	97	4.21	1.94	2.31	100/100	–	–
**P1**	91	4.74	3.23	1.61	85/84.6	8/8.7	3/7.8
**P1c**	95	4.32	2.00	2.31	83/83.4	8/8.9	6/7.8
**P2**	89	4.92	2.42	1.55	61/61.2	20/24.4	9/14.5
**P2c**	97	5.45	1.87	2.91	60/60.0	24/23.9	16/16.0

^1^ SEC data (THF, polystyrene standard). ^2^ A rough estimation by integration of the signals in ^13^C {^1^H} NMR spectra/calculated on the basis of α-olefin ratios in mixtures **M1**–**M2c**.

## Data Availability

The data presented in this study are available on request from the corresponding author.

## Supplementary Materials

The following are available online at https://www.mdpi.com/article/10.3390/polym13223930/s1, Figure S1: ^1^H NMR spectrum (400 MHz, CDCl_3_, 20 °C) of 2-ethyl-2-(methoxymethyl)hexan-1-ol (main fraction, B. p. 90–110 °C/16 mbar), Figure S2: ^1^H NMR spectrum (400 MHz, CDCl_3_, 20 °C) of BMMH, Figure S3: ^13^C{^1^H} NMR spectrum (101 MHz, CDCl_3_, 20 °C) of BMMH, Figure S4: Reference ^13^C {^1^H} NMR spectra (CDCl_3_, 50 °C) of isotactic poly(1-hexene), poly(1-octene) and poly(1-decene) (top, middle and bottom, respectively), Figure S5: ^1^H NMR spectrum (CDCl_3_, 50 °C) of **P1**, Figure S6: ^1^H NMR spectrum (CDCl_3_, 50 °C) of **P1c**, Figure S7: ^1^H NMR spectrum (CDCl_3_, 50 °C) of **P2**, Figure S8: ^1^H NMR spectrum (CDCl_3_, 50 °C) of **P2c**, Figure S9: ^13^C {^1^H} NMR spectrum (CDCl_3_, 50 °C) of **P1**, Figure S10: ^13^C {^1^H} NMR spectrum (CDCl_3_, 50 °C) of **P1c**, Figure S11: ^13^C {^1^H} NMR spectrum (CDCl_3_, 50 °C) of **P2**, Figure S12: ^13^C {^1^H} NMR spectrum (CDCl_3_, 50 °C) of **P2c**.
