# Supplementary material for: Tandem Synthesis of Ultra-High Molecular Weight Drag Reducing Poly-α-Olefins for Low-Temperature Pipeline Transportation
Source: Polymers (Basel). 2021 Nov 14;13(22):3930. doi: 10.3390/polym13223930 (PMC8621585; doi:10.3390/polym13223930)
Supplement: Supplementary file 1 [file polymers-13-03930-s001.zip › polymers-1457732-supplementary.pdf]

Tandem Synthesis of Ultra-High Molecular Weight Drag Reducing Poly-α-olefins for Low-temperature Pipeline Transportation

Ilya E. Nifant'ev[1,2,*], Alexander N. Tavtorkin[1], Alexey A. Vinogradov[2], Sofia A. Korchagina[1], Maria S. Chinova[1], Roman S. Borisov[2], Grigory A. Artem'ev[3] and Pavel V. Ivchenko[1,2]

[1] A.V. Topchiev Institute of Petrochemical Synthesis RAS, 29 Leninsky Pr., 119991 Moscow, Russia; tavtorkin@yandex.ru (A.T.); korchagina@ips.ac.ru (S.K.); chinova@yandex.ru (M.C.); phpasha1@yandex.ru (P.I.).

[2] Chemistry Department, M.V. Lomonosov Moscow State University, 1–3 Leninskie Gory, 119991 Moscow, Russia; inif@org.chem.msu.ru (I.N.); inpv@org.chem.msu.ru (P.I.).

[3] I.Ya. Postovsky Institute of Organic Synthesis, Ural Division of RAS, 22 S. Kovalevskoy Str., Yekaterinburg, Russia.

* Correspondence: ilnif@yahoo.com

Supplementary Information

S1. Titanium-magnesium ZN catalyst S2–S4

S2. Polymer characteristics S5–S9

S1. Titanium-magnesium ZN catalyst

S1.1. Synthesis of donor BMMH: NMR spectra

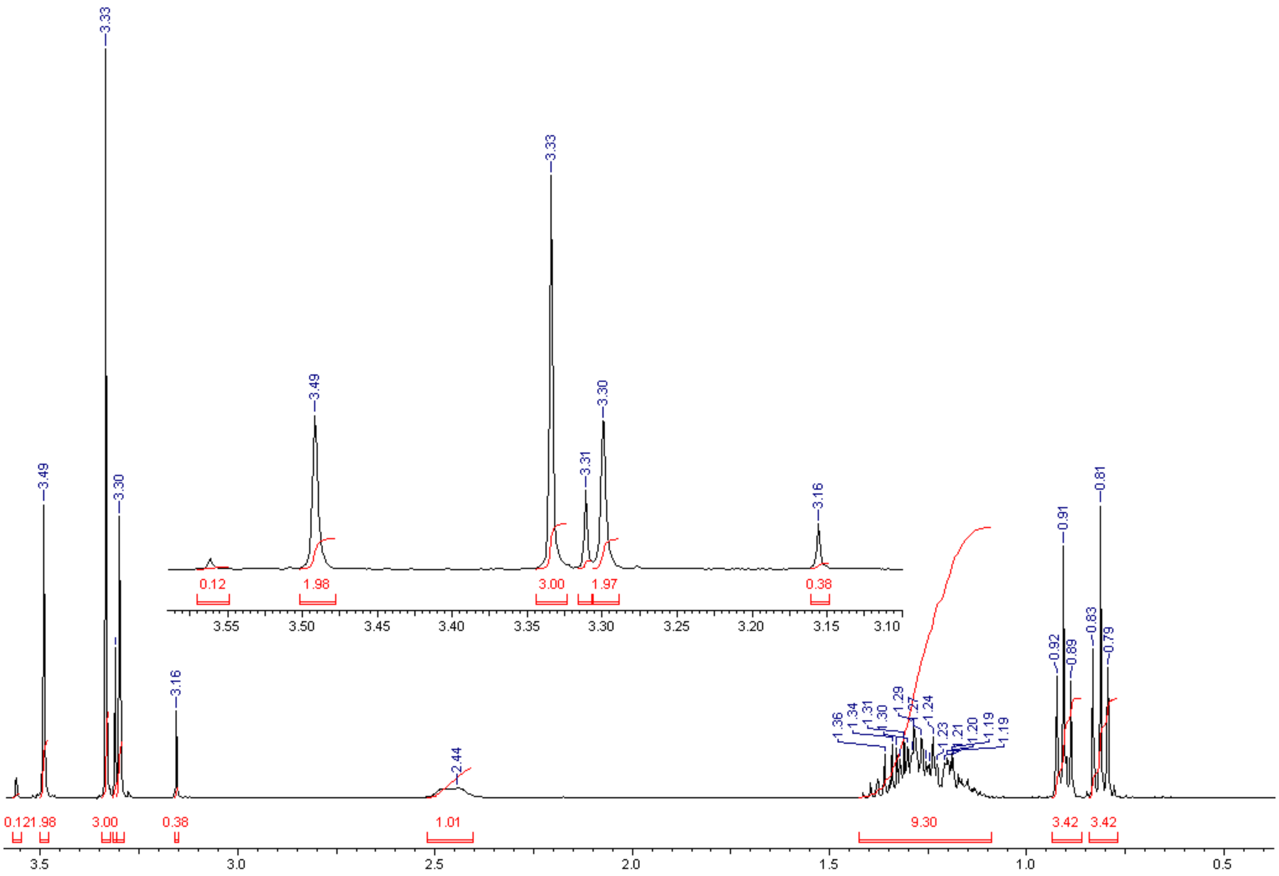

Figure S1. ^{1}H NMR spectrum (400 MHz, $CDCl_3$, 20 °C) of 2-ethyl-2-(methoxymethyl)hexan-1-ol (main fraction, B. p. 90-110 °C/16 mbar).

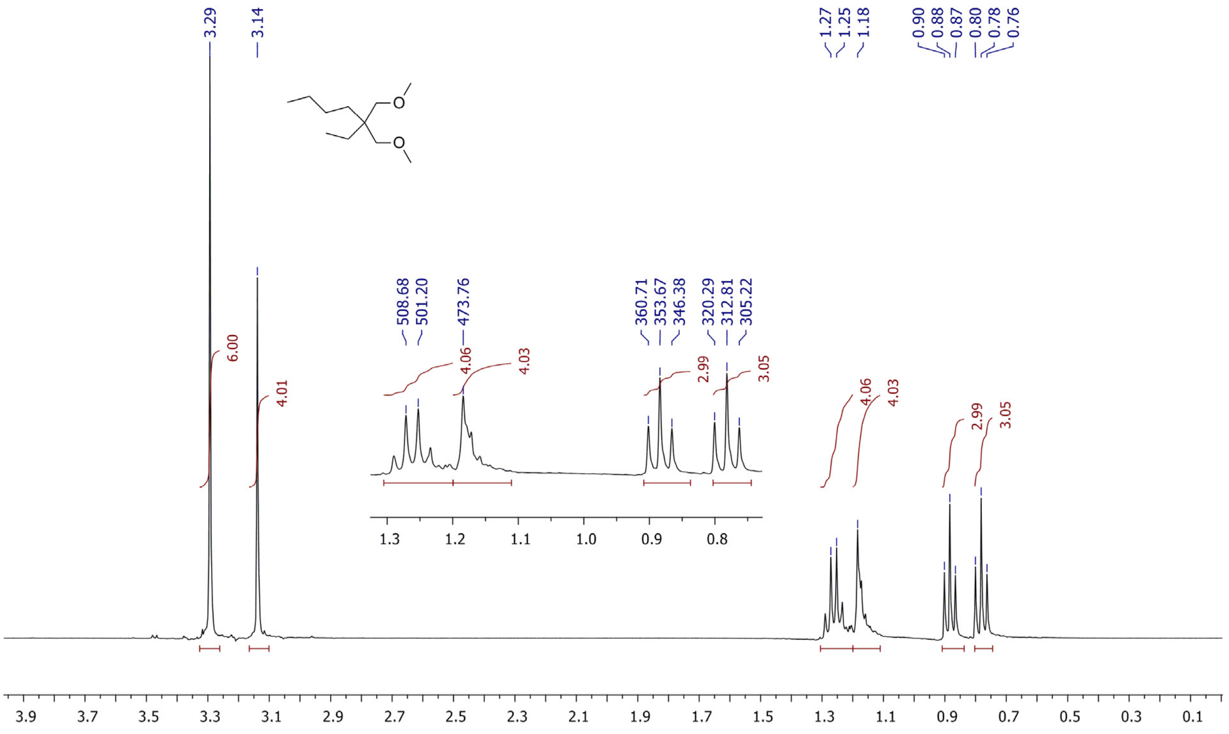

Figure S2. ^{1}H NMR spectrum (400 MHz, $CDCl_3$, 20 °C) of BMMH.

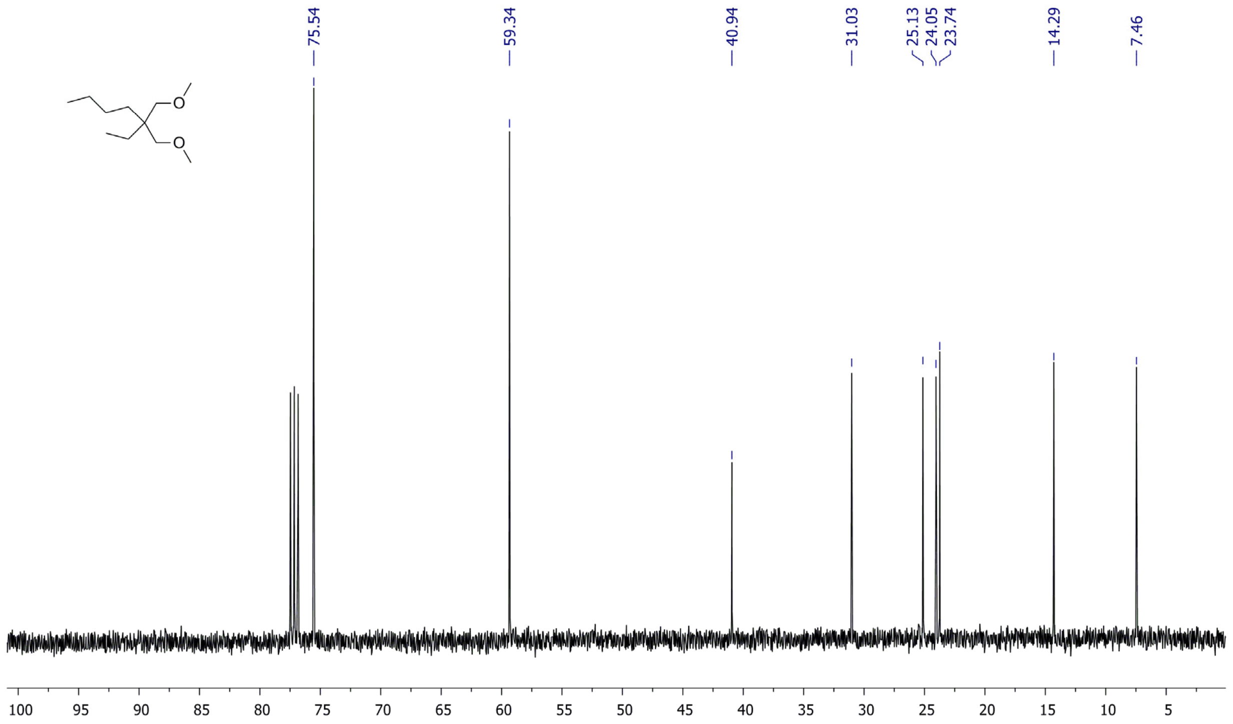

Figure S3. $^{13}C\{^1H\}$ NMR spectrum (101 MHz, $CDCl_3$, 20 °C) of BMMH.

S1.2. Synthesis of TMC

S1.2.1. Equipment

For the synthesis of magnesium ethoxide ($Mg(OEt)_2$) and TMC, we used 15 L and 10 L glass jacketed reactor units, respectively (Ablaze Glass Works Pvt. Ltd., Vedodara, India). Each unit was equipped with mechanic stirrer, distillation column with condenser, thermostate, and measuring vessels for reagent loading. Argon (99.999%, Linde Gas, Russian Federation) was used to provide inert atmosphere.

S1.2.2. Synthesis of magnesium ethoxide

Magnesium turnings (531 g, 21.85 mol) NH_4Cl (5.0 g) and dry EtOH (1.5 L) were placed into the reactor. The mixture was heated to 60 °C with heating, and after the beginning of the violent process with intensive hydrogen evolution, EtOH (8.0 L) was added within 2 h with the maintenance of the reaction temperature in the range of 70–75 °C. Upon completion of the addition of EtOH, the mixture was stirred at 75 °C to complete the reaction that was detected by the absence of hydrogen evolution. The reactor was cooled, toluene (5 L) was added, and the mixture was heated to 110 °C with removing EtOH by distillation. The suspension of $Mg(OEt)_2$ was than used in the synthesis of TMC.

S1.2.3. Synthesis of TMC, addition of $TiCl_4$ to $Mg(OEt)_2$ (**TMC-1**)

$Mg(OEt)_2$ (0.5 kg, 2.5 L of suspension in toluene) was loaded to reactor, the mixture was heated to 60 °C. The solution of $TiCl_4$ (0.25 L) in toluene (2.5 L) was added; the temperature of the reaction mixture raised to 104 °C. After cooling to 90 °C, the solution containing $TiCl_4$ (1.25 L) and toluene (0.25 L) was added within 1 h. Next, BMMH (480 mL) was added, and the mixture was refluxed with stirring for 4 h. The stirring was stopped, and the mixture was allowed to cool to 20 °C. After 2 h, clear layer was separated using siphon, and toluene (4 L) was added. The mixture was heated to 50 °C, and stirred intensively within 10 min. After the precipitation of the TMC (20 h), clear layer was separated using siphon, and solution containing $TiCl_4$ (0.5 L) and toluene (0.75 L) was added. The mixture was heated to reflux, and after 3 h of stirring the heating was stopped. After 30 min, the stirring was also stopped, and the mixture was allowed to cool to 25 °C. After 12 h, clear layer was separated using siphon, toluene (4 L) was added with stirring, the mixture was heated to 45 °C and stirred for 30 min.

After cooling to 20 °C, the mixture was left alone for 12 h. Clear layer was separated using siphon, *n*-heptane (4 L) was added, the mixture was heated to 45 °C and stirred within 10 min. Such flushing with *n*-heptane was repeated four times. At the final stage, *n*–heptane was added with stirring to reach the total volume of the catalyst dispersion of 5 L. This suspension was then analyzed and used in polymerization experiments.

S1.2.4. Synthesis of TMC, addition of $Mg(OEt)_2$ to $TiCl_4$ (**TMC-2**)

$TiCl_4$ (2.7 L) and toluene (1.25 L) were loaded to reactor. After heating to 60 °C with stirring, $Mg(OEt)_2$ (1.0 kg, 5.0 L of suspension in toluene) was added dropwise. BMMH (235 mL) was then added, and the mixture was refluxed within 4 h. The following procedure of the preparation of TMC suspeision was completely analogous to the procedure described in Section S1.2.3.

S1.3. Analyzing and testing of TMC

S1.3.1. Determination of Ti content

A sample of the TMC (~100 mg) was placed into a 50 mL volumetric flask. 6 M HNO_3 (10 mL) was added, and the flask was filled with distilled water to a calibration mark. The mixture was shaken for 10 min, and 5 mL solution probe was transferred into the titration flask. The solution of 0.01 M disodium EDTA (5 mL) and methyl red as an indicator (1-2 drops of 1% ethanol solution) were added (causing the red color of the solution). Then, hexamethylenetetramine was added by small portions until the indicator changed color to light yellow. After that, the indicator xylenol orange (2–3 drops of 1% ethanol solution) was added (causing the bright yellow color of the solution), and the excess EDTA was titrated by $Pb(NO_3)_2$ until the indicator changed color to wine red. The calculations of n_{Ti} (Ti content in mmol) and MC_{Ti} (Ti mass content, %) were made using formula S1 and S2.

$$n_{\mathrm{Ti}} = \frac{(V_{\mathrm{b}} - V_{\mathrm{exp}}) \cdot 0.50}{5} \quad \text{(S1)}$$

where V_b is the volume of the $Pb(NO_3)_2$ solution consumed in the blank probe titration; V_{exp} is the volume of $Pb(NO_3)_2$ solution consumed in the titration of the catalyst sample.

$$MC_{\mathrm{Ti}} = \frac{n_{\mathrm{Ti}} \cdot 47.87}{m_{\mathrm{TMC}}} \cdot 100\% \quad \text{(S2)}$$

where m_{TMC} is a weight of the sample of TMC.

Molar concentration of Ti in TMC suspension [Ti] (mol/L) was calculated by the formula (S3).

$$[Ti] = \frac{n_{\mathrm{Ti}}}{V_{TMC}} \quad \text{(S3)}$$

where V_{TMC} is the volume of the probe of the TMC suspension. For **TMC-1** and **TMC-2** the values of [Ti] were 0.08 and 0.16 M, respectively.

S1.3.2. Estimation of the catalytic activity

1-Hexene (20 mL) was placed into 50 mL vial, TIBA (0.4 mL of 1M solution in heptane) was added. The vial was cooled to –40 °C, TMC suspension (8 μL) was added with stirring, and the mixture was stored at –12 °C within 10 days. The yields of poly(1-hexene) were 88% and 97%, M_n values were found to be $1.98 \cdot 10^6$ and $1.94 \cdot 10^6$ Da for **TMC-1** and **TMC-2**, respectively. On the basis of the TMC activity, **TMC-2** was used in (co)polymerization experiments.

S2. Polymer characteristics

S2.1. Reference ^{13}C NMR spectra

^{13}C NMR spectra of isotactic UHMW homopolymers of C6, C8 and C10 linear α-olefins are presented in Figure S4. These spectra were used as the basis for the schematic spectra presented in Figure 2a.

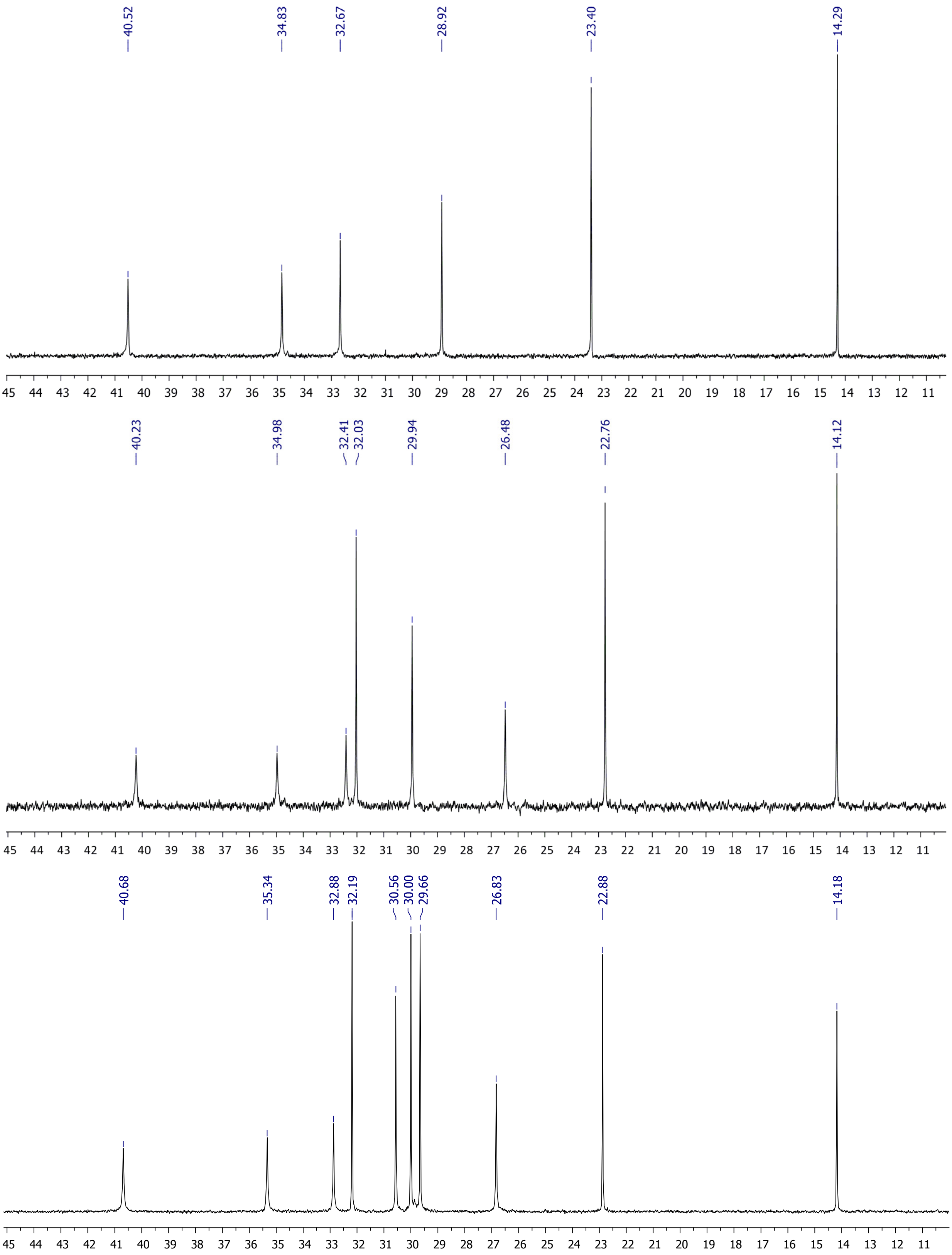

Figure S4. Reference ^{13}C {^{1}H} NMR spectra ($CDCl_3$, 50 °C) of isotactic poly(1-hexene), poly(1-octene) and poly(1-decene) (top, middle and bottom, respectively).

S2.2. ^{1}H NMR spectra of copolymers

The samples were prepared by dissolution of 10 mg of copolymer in $CHCl_3$ (2 mL), evaporation of the solvent under reduced pressure, and drying for 24 h in vacuo (0.013 mbar). ^{1}H NMR spectra of copolymers **P1**—**P2c** are presented in Figures S5—S8. The spectra of **P1** and **P2** demonstrate the presence of the inactive olefins (5-methylenenonane, internal decenes) and low-active α-olefins (4-vinyloctane, 4-ethyl-1-octene).

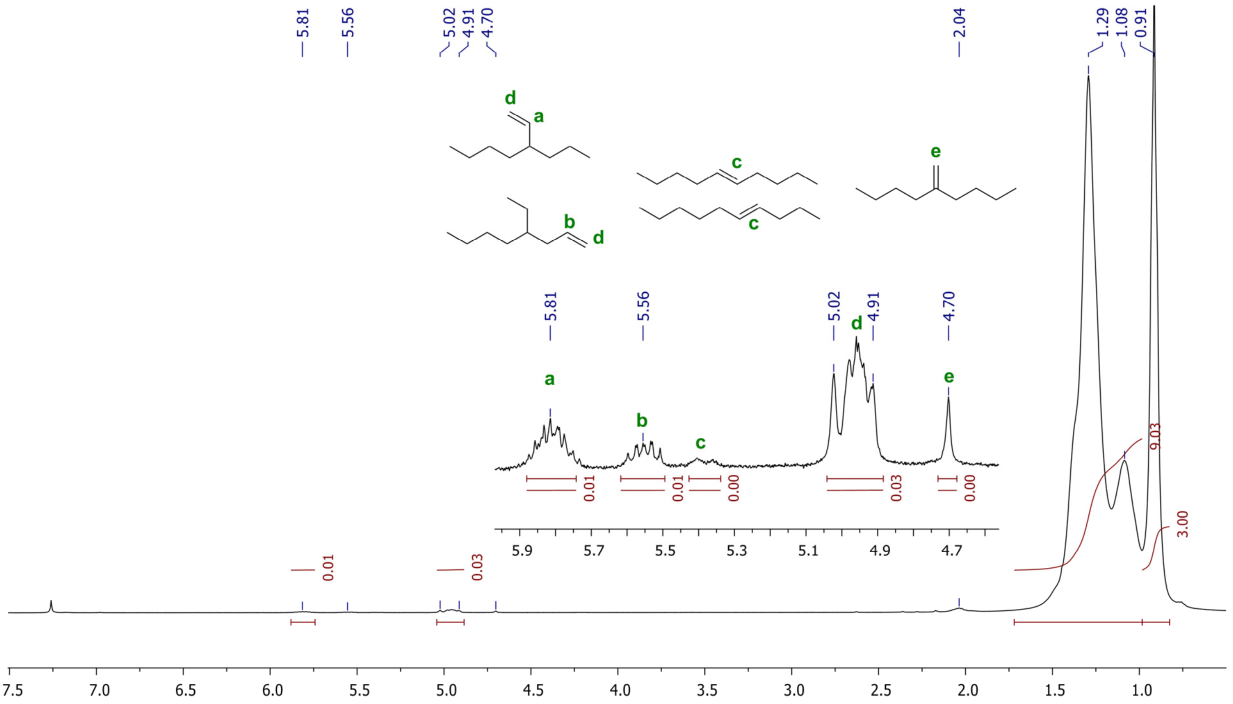

Figure S5. ^{1}H NMR spectrum ($CDCl_3$, 50 °C) of **P1**.

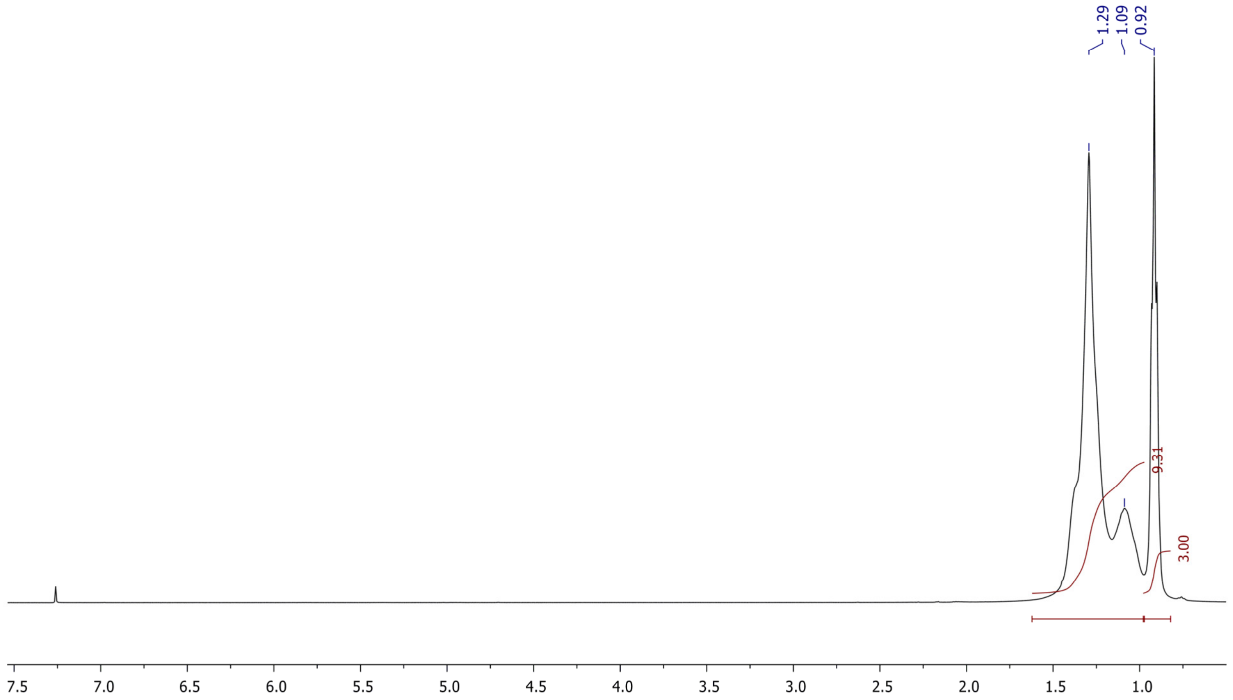

Figure S6. ^{1}H NMR spectrum ($CDCl_3$, 50 °C) of **P1c**.

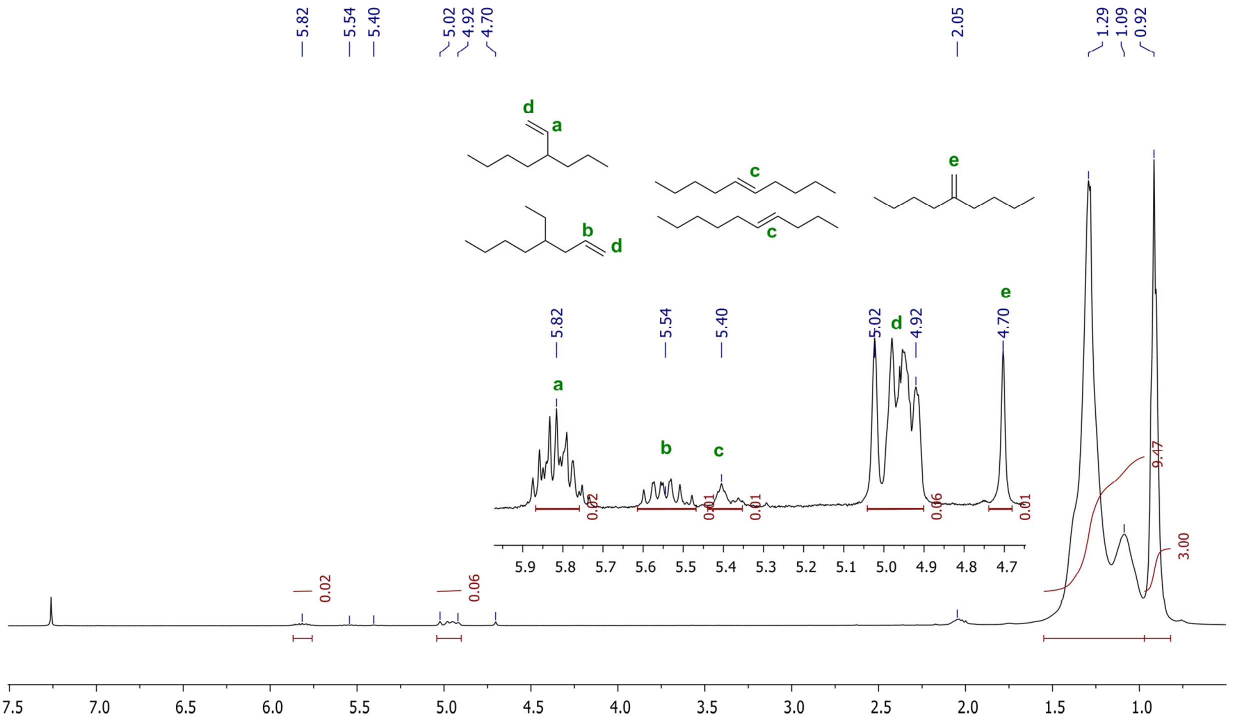

Figure S7. ^{1}H NMR spectrum ($CDCl_3$, 50 °C) of **P2**.

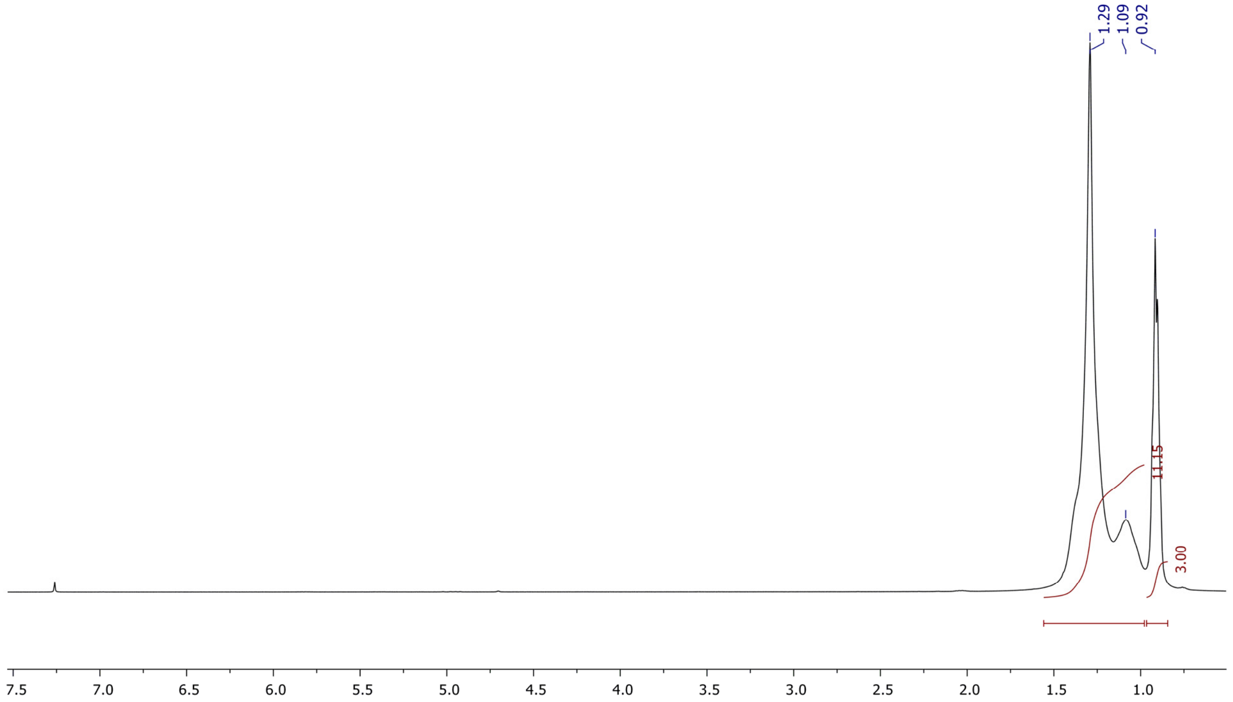

Figure S8. ^{1}H NMR spectrum ($CDCl_3$, 50 °C) of **P2c**.

S2.3. ^{13}C NMR spectra of copolymers

To eliminate unreacted olefins, the samples were prepared by dissolution of 10 mg of copolymer in $CHCl_3$ (1 mL), slow addition of the solution to 20 mL of Et_2O with stirring, separation of the copolymer by decantation, and drying for 24 h in vacuo (0.013 mbar). ^{13}C NMR spectra of copolymers **P1**—**P2c** are presented in Figures S9—S12.

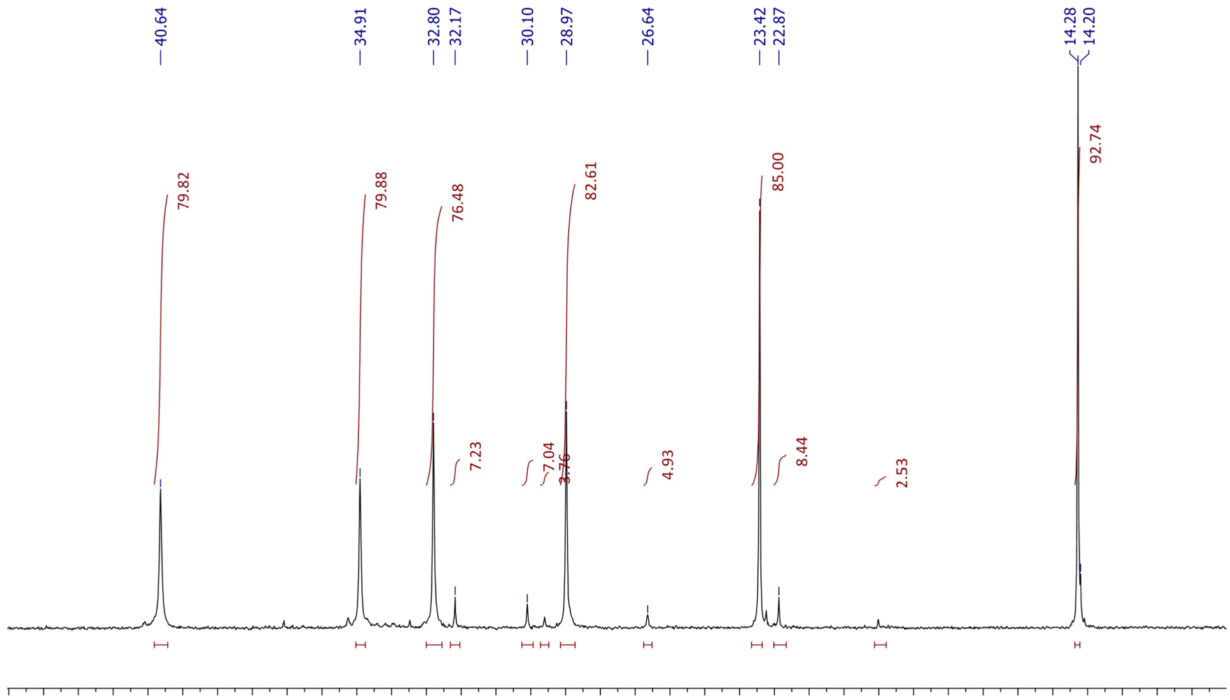

Figure S9. ^{13}C {^{1}H} NMR spectrum ($CDCl_3$, 50 °C) of **P1**.

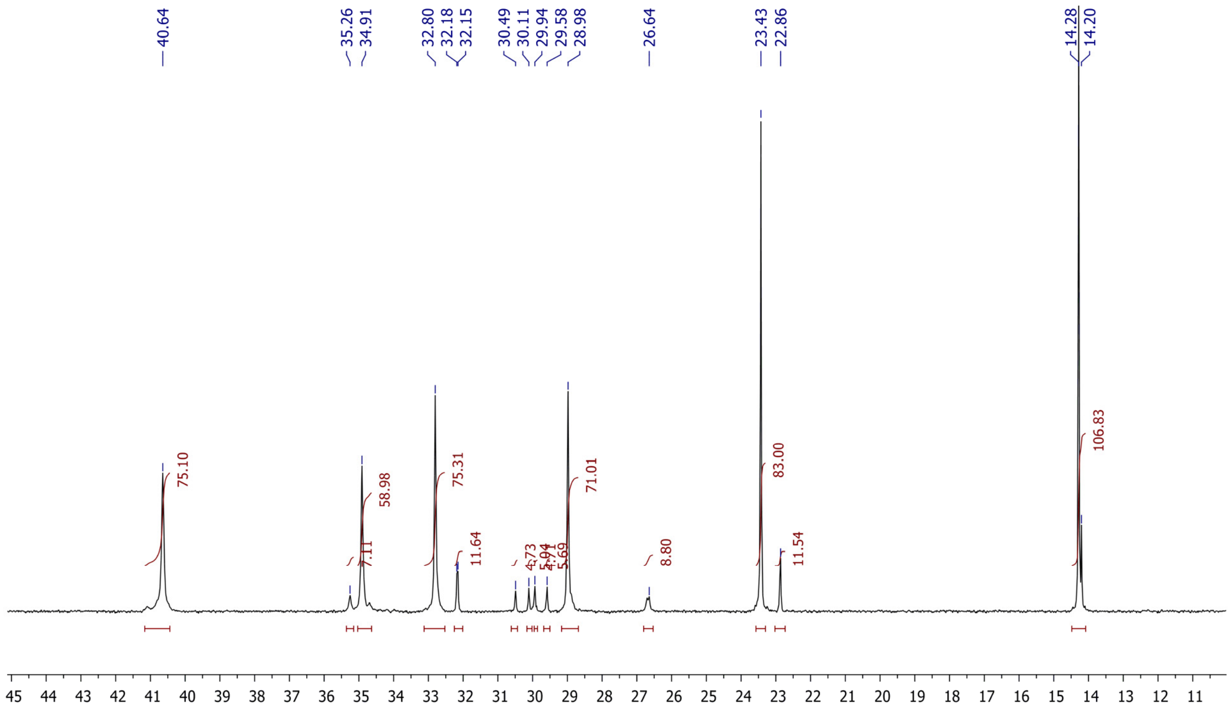

Figure S10. ^{13}C {^{1}H} NMR spectrum ($CDCl_3$, 50 °C) of **P1c**.

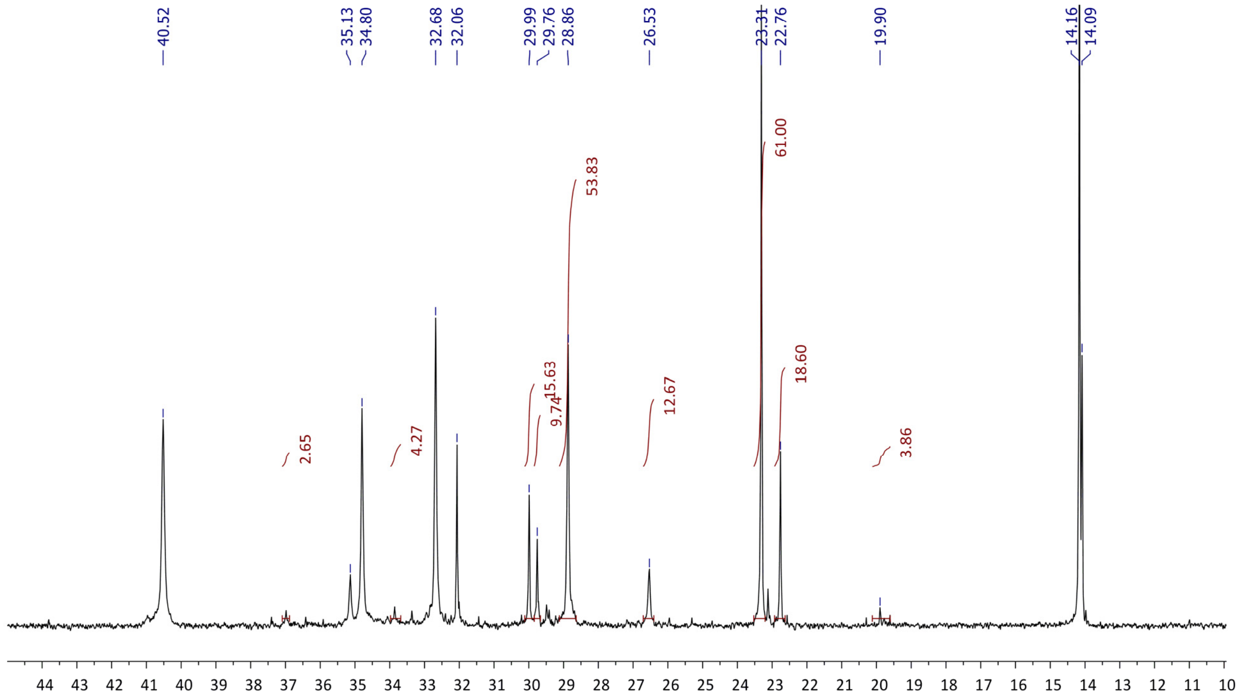

Figure S11. ^{13}C {^1H} NMR spectrum ($CDCl_3$, 50 °C) of **P2**.

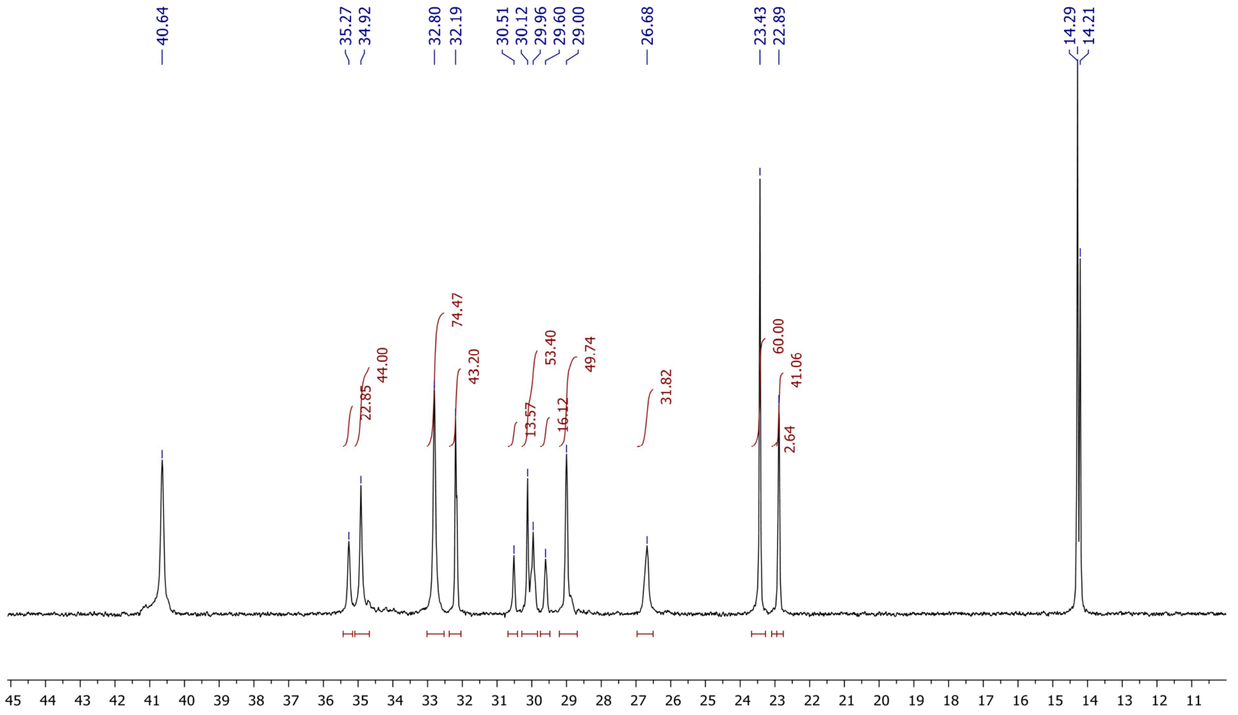

Figure S12. ^{13}C {^1H} NMR spectrum ($CDCl_3$, 50 °C) of **P2c**.
